# Supplementary material for: Regulation of pDC fate determination by histone deacetylase 3
Source: eLife. 2023 Nov 27;12:e80477. doi: 10.7554/eLife.80477 (PMC10732571; doi:10.7554/eLife.80477)
Supplement: Figure 1—figure supplement 2—source data 1. [file elife-80477-fig1-figsupp2-data1.zip › Figure 1- figure supplement 2D source data 1/2. Labeled files/Figure 1- figure supplement 2D source data 1.docx]

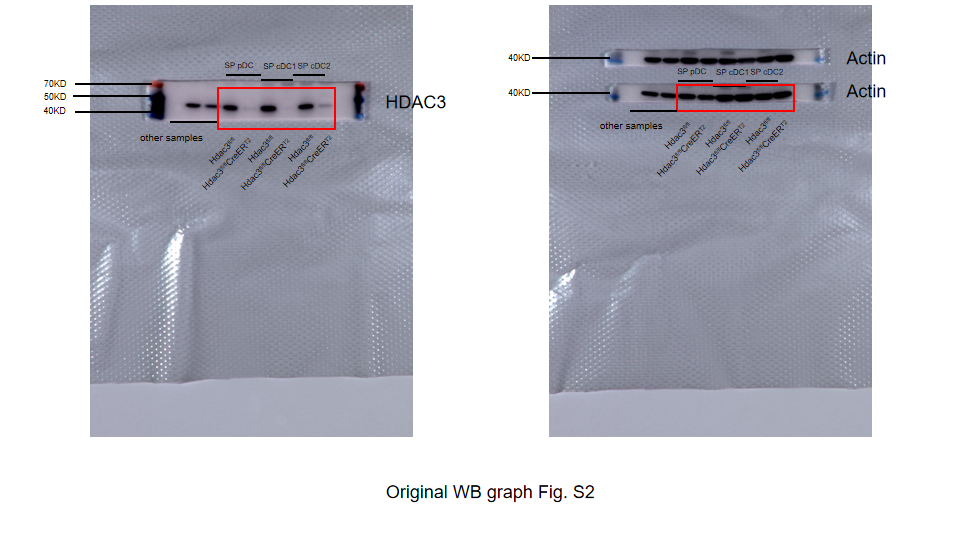


Another gel

**Figure 1 - figure supplement 2D - source data 1.** Western blot of HDAC3 knockout efficiency in *Rosa26*-CreER^T2^ induce HDAC3 conditional knockout splenic DC subsets.
