# Supplementary figures and images for: Regulation of pDC fate determination by histone deacetylase 3
Source: eLife. 2023 Nov 27;12:e80477. doi: 10.7554/eLife.80477 (PMC10732571; doi:10.7554/eLife.80477)

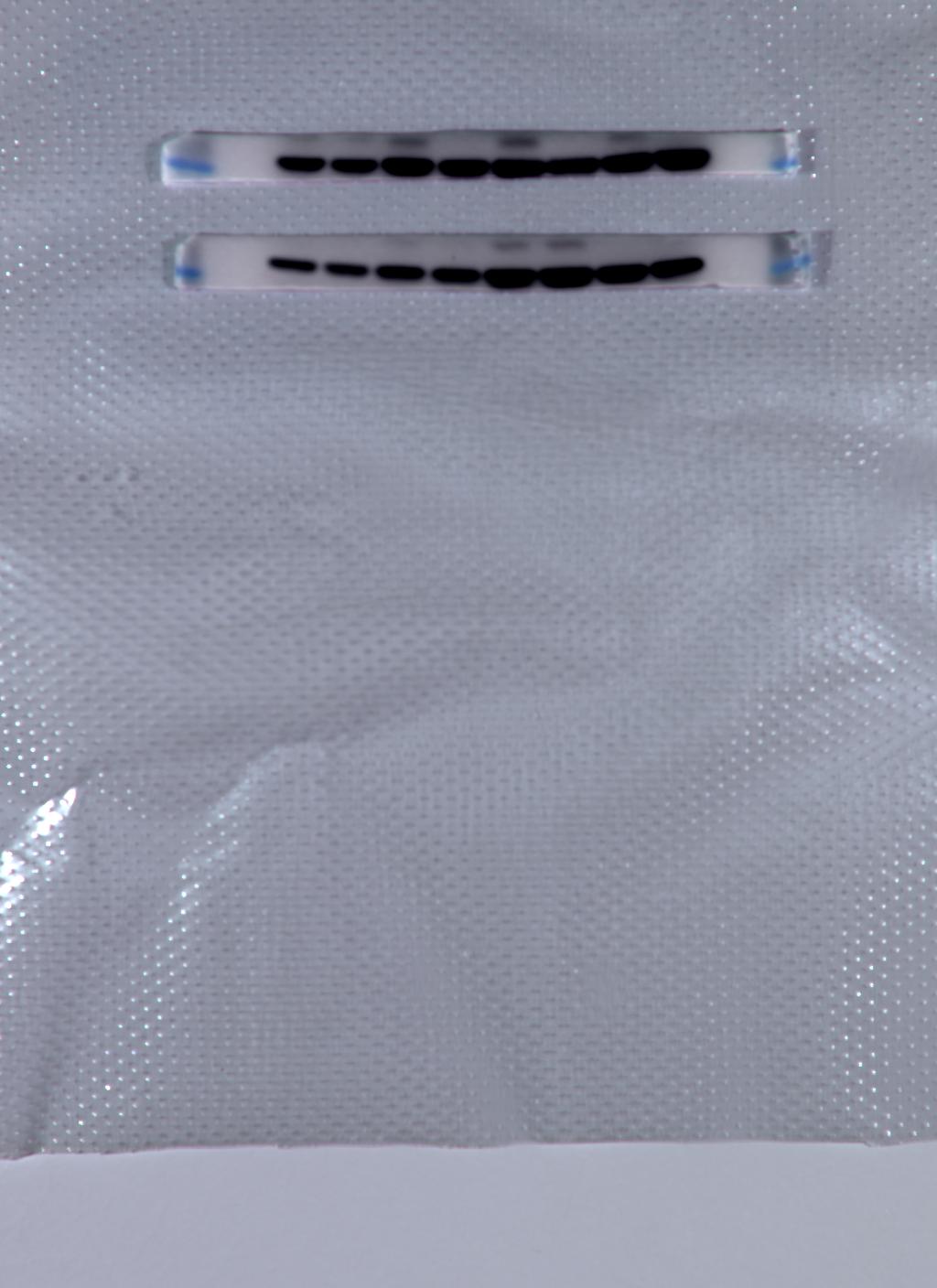

Supplement: Figure 1—figure supplement 2—source data 1. [file elife-80477-fig1-figsupp2-data1.zip › Figure 1- figure supplement 2D source data 1/1. Original files/Actin/191014 act12 s2 2019.10.14_10.48.16_Ch+Marker.jpg]

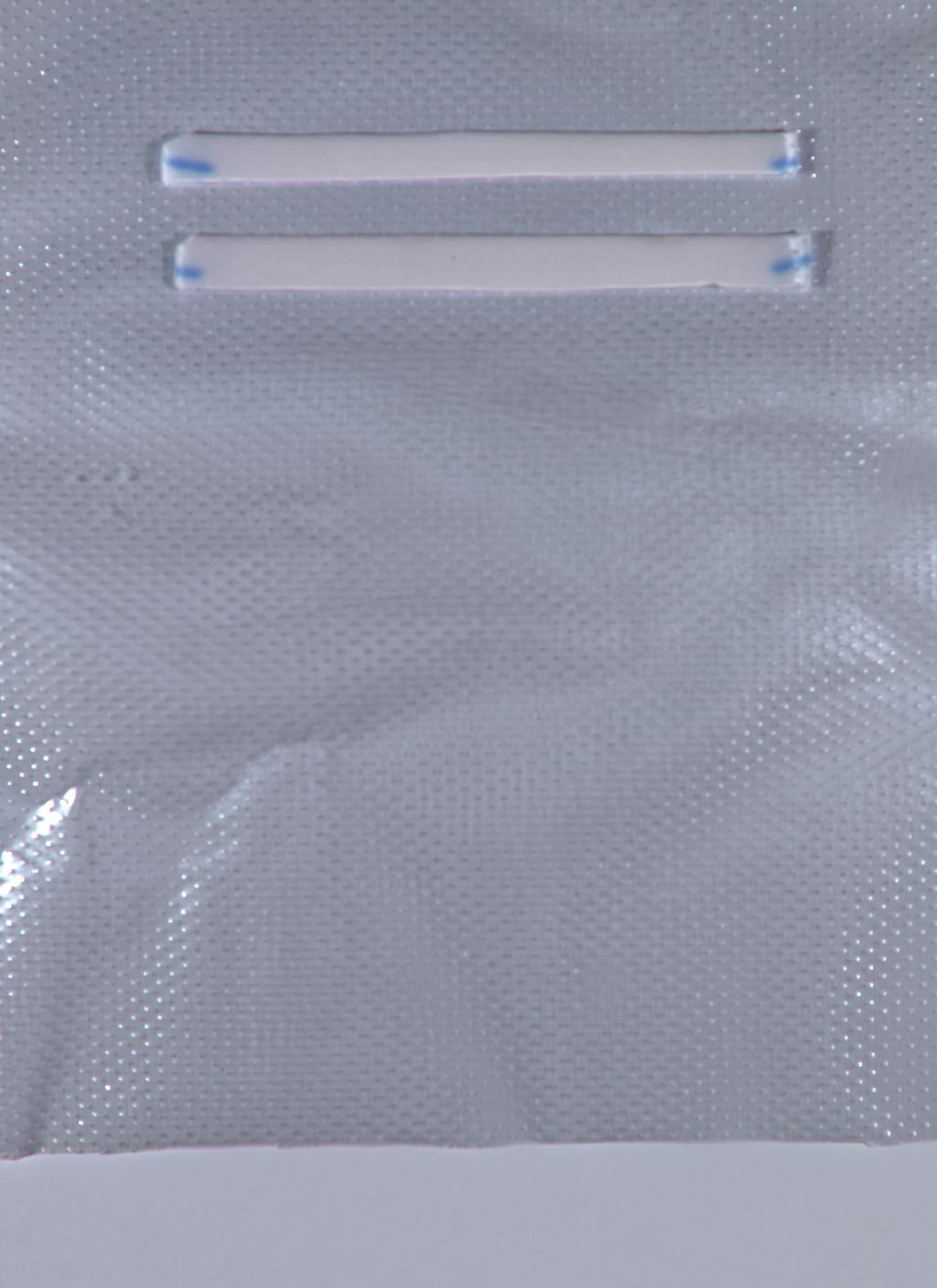

Supplement: Figure 1—figure supplement 2—source data 1. [file elife-80477-fig1-figsupp2-data1.zip › Figure 1- figure supplement 2D source data 1/1. Original files/Actin/191014 act12 s2 2019.10.14_10.48.16_Ch-Marker.jpg]

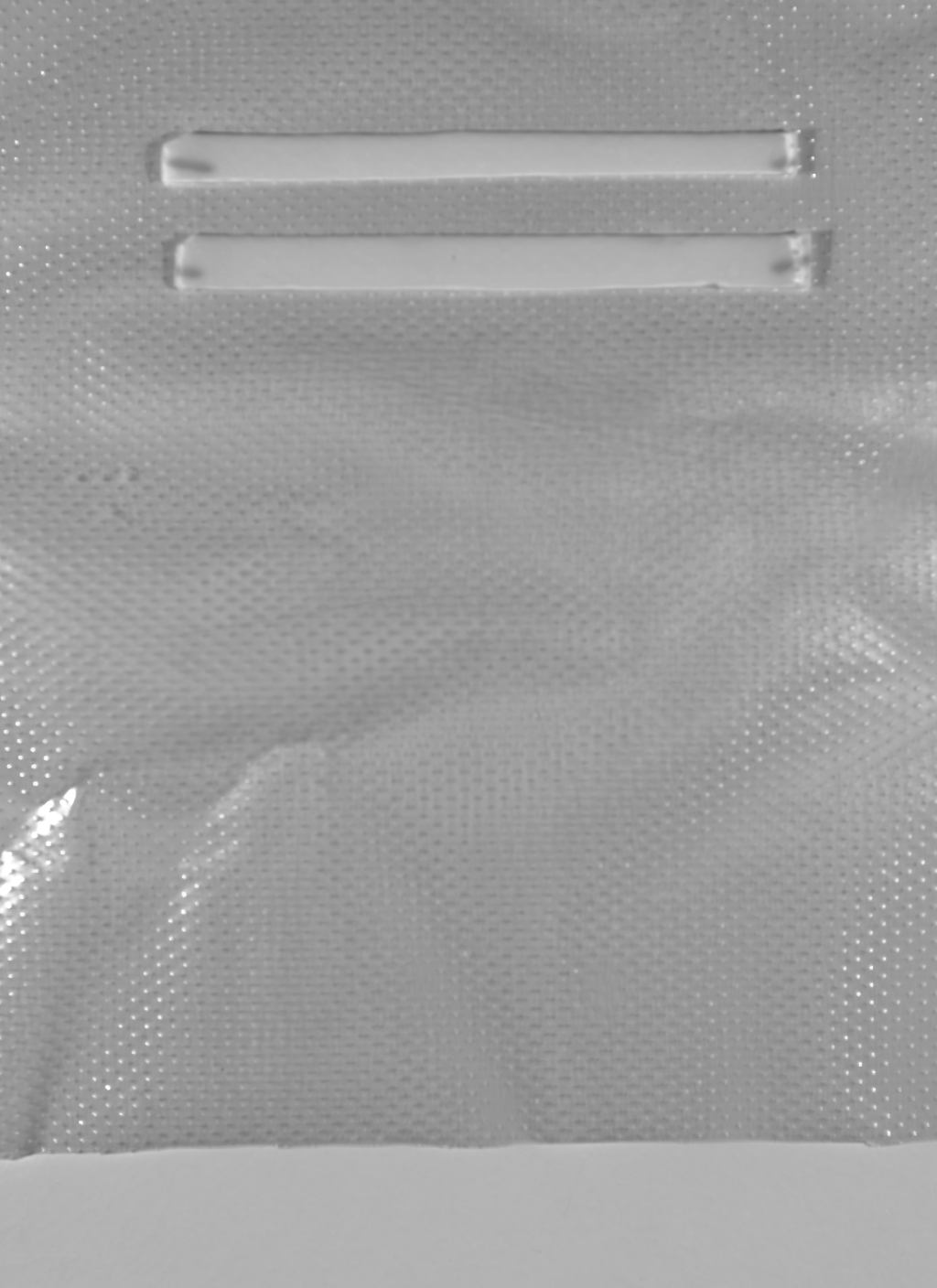

Supplement: Figure 1—figure supplement 2—source data 1. [file elife-80477-fig1-figsupp2-data1.zip › Figure 1- figure supplement 2D source data 1/1. Original files/Actin/191014 act12 s2 2019.10.14_10.48.16_Ch-Marker.tif]

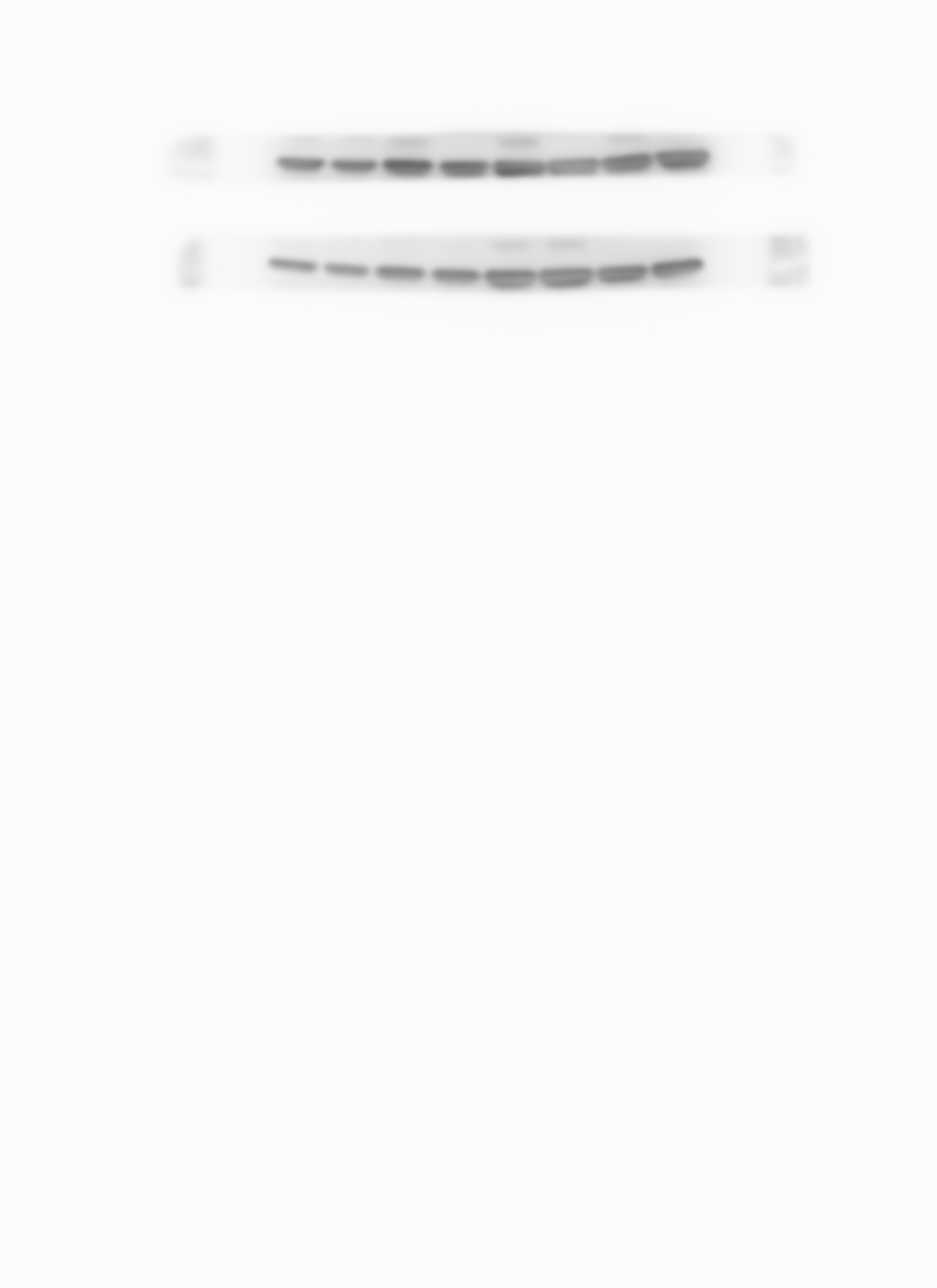

Supplement: Figure 1—figure supplement 2—source data 1. [file elife-80477-fig1-figsupp2-data1.zip › Figure 1- figure supplement 2D source data 1/1. Original files/Actin/191014 act12 s2 2019.10.14_10.48.16_Ch.tif]

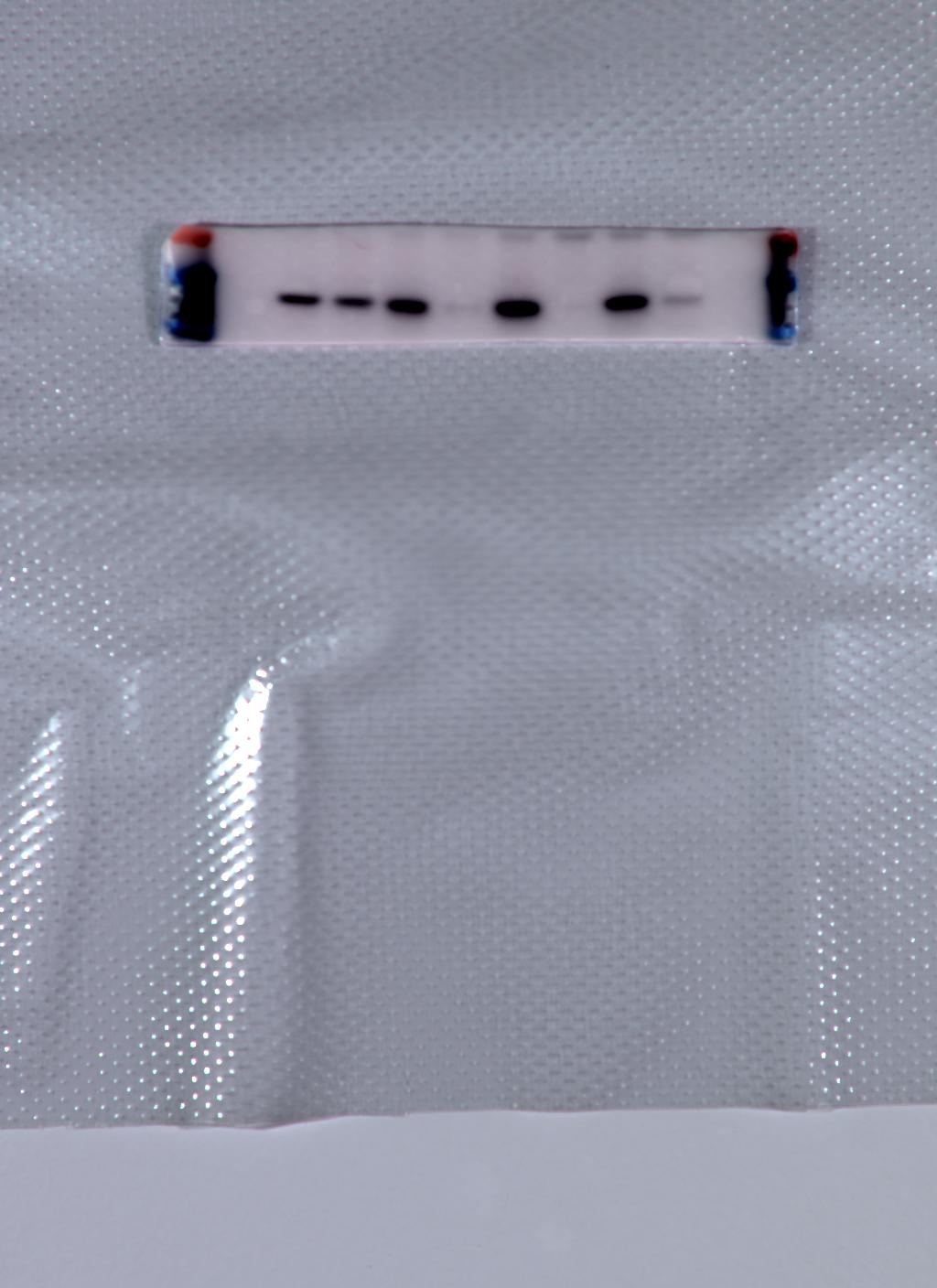

Supplement: Figure 1—figure supplement 2—source data 1. [file elife-80477-fig1-figsupp2-data1.zip › Figure 1- figure supplement 2D source data 1/1. Original files/HDAC3/191013 h3 s18 2019.10.13_15.11.08_Ch+Marker.jpg]

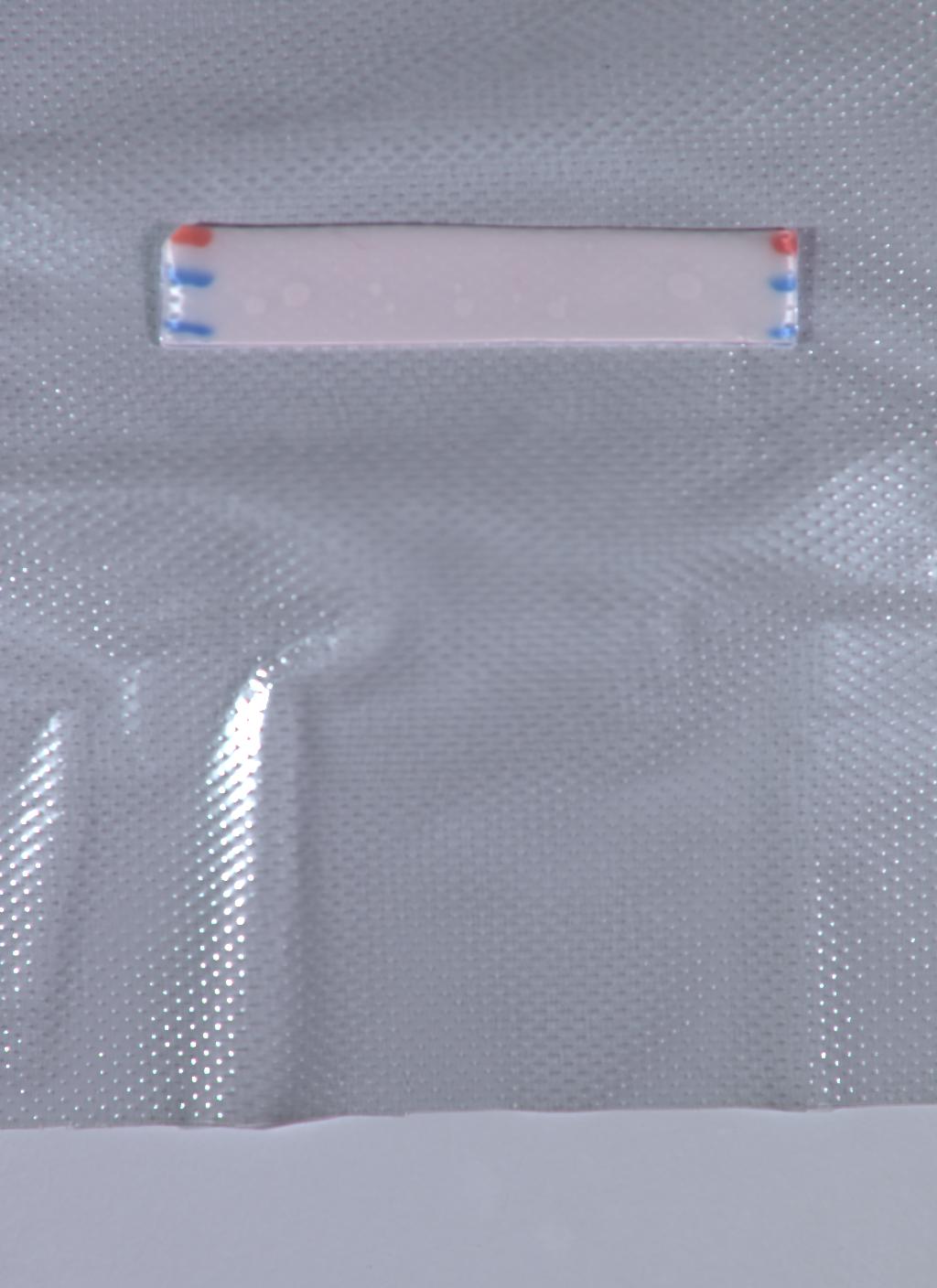

Supplement: Figure 1—figure supplement 2—source data 1. [file elife-80477-fig1-figsupp2-data1.zip › Figure 1- figure supplement 2D source data 1/1. Original files/HDAC3/191013 h3 s18 2019.10.13_15.11.08_Ch-Marker.jpg]

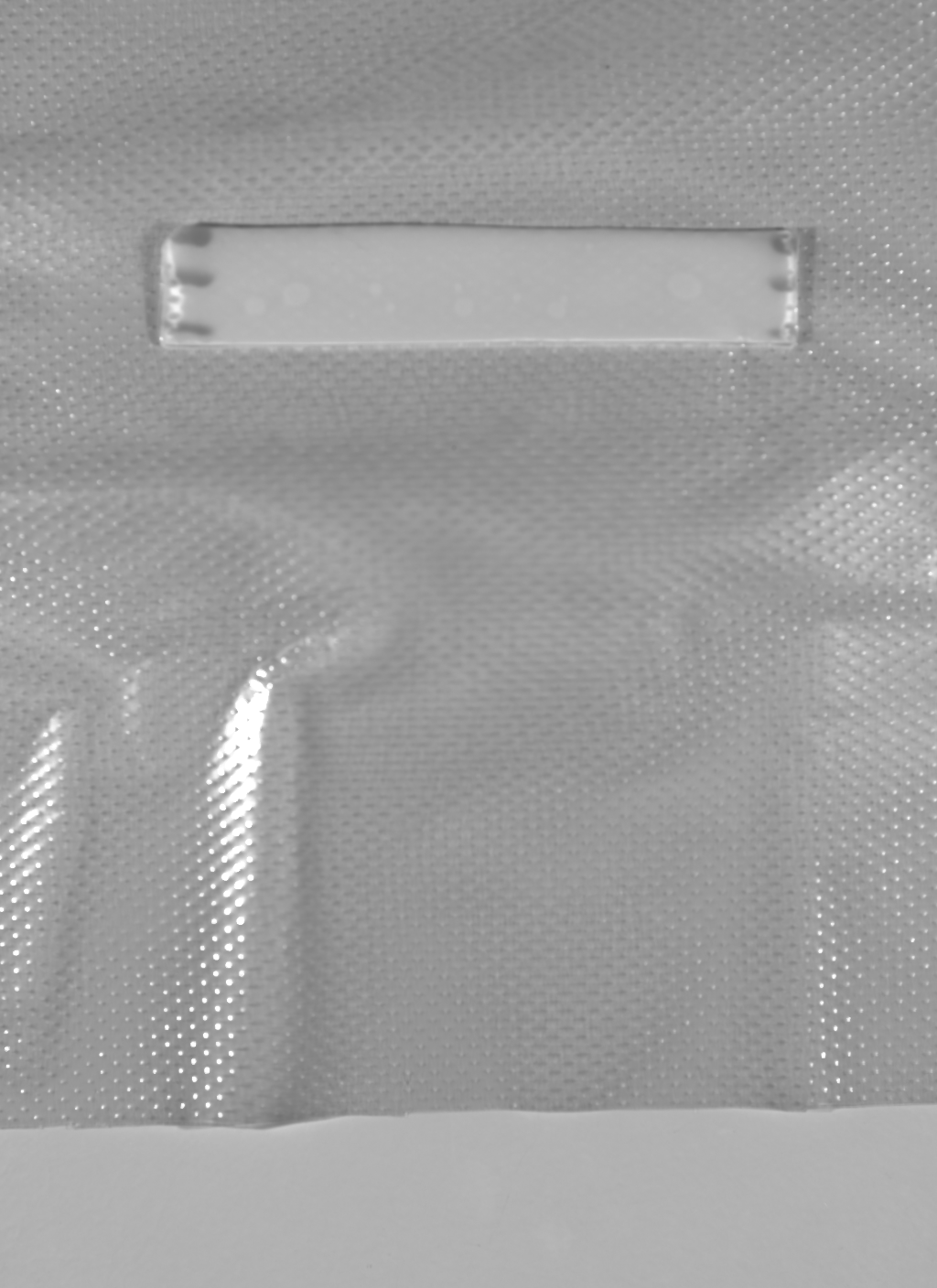

Supplement: Figure 1—figure supplement 2—source data 1. [file elife-80477-fig1-figsupp2-data1.zip › Figure 1- figure supplement 2D source data 1/1. Original files/HDAC3/191013 h3 s18 2019.10.13_15.11.08_Ch-Marker.tif]

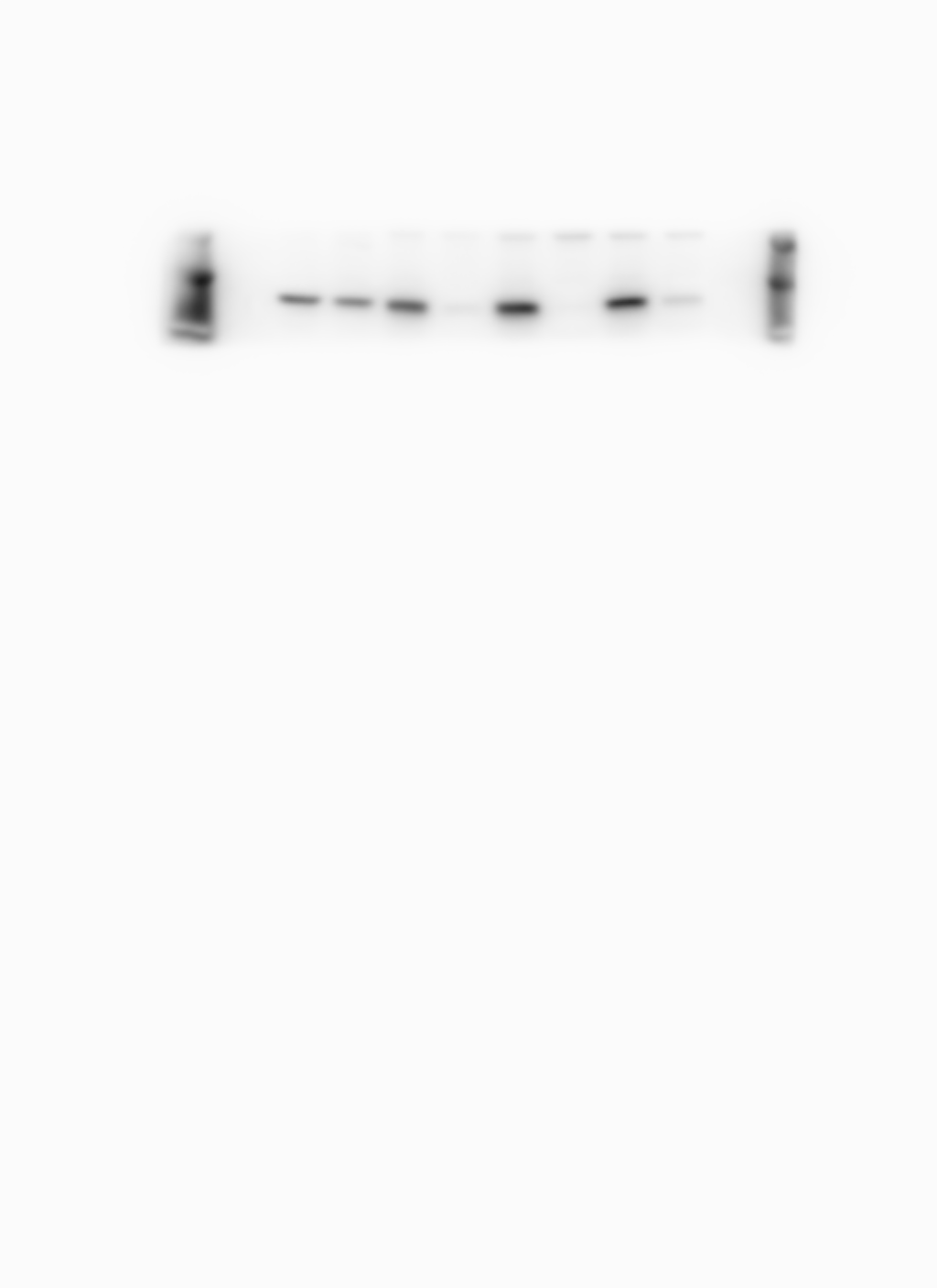

Supplement: Figure 1—figure supplement 2—source data 1. [file elife-80477-fig1-figsupp2-data1.zip › Figure 1- figure supplement 2D source data 1/1. Original files/HDAC3/191013 h3 s18 2019.10.13_15.11.08_Ch.tif]

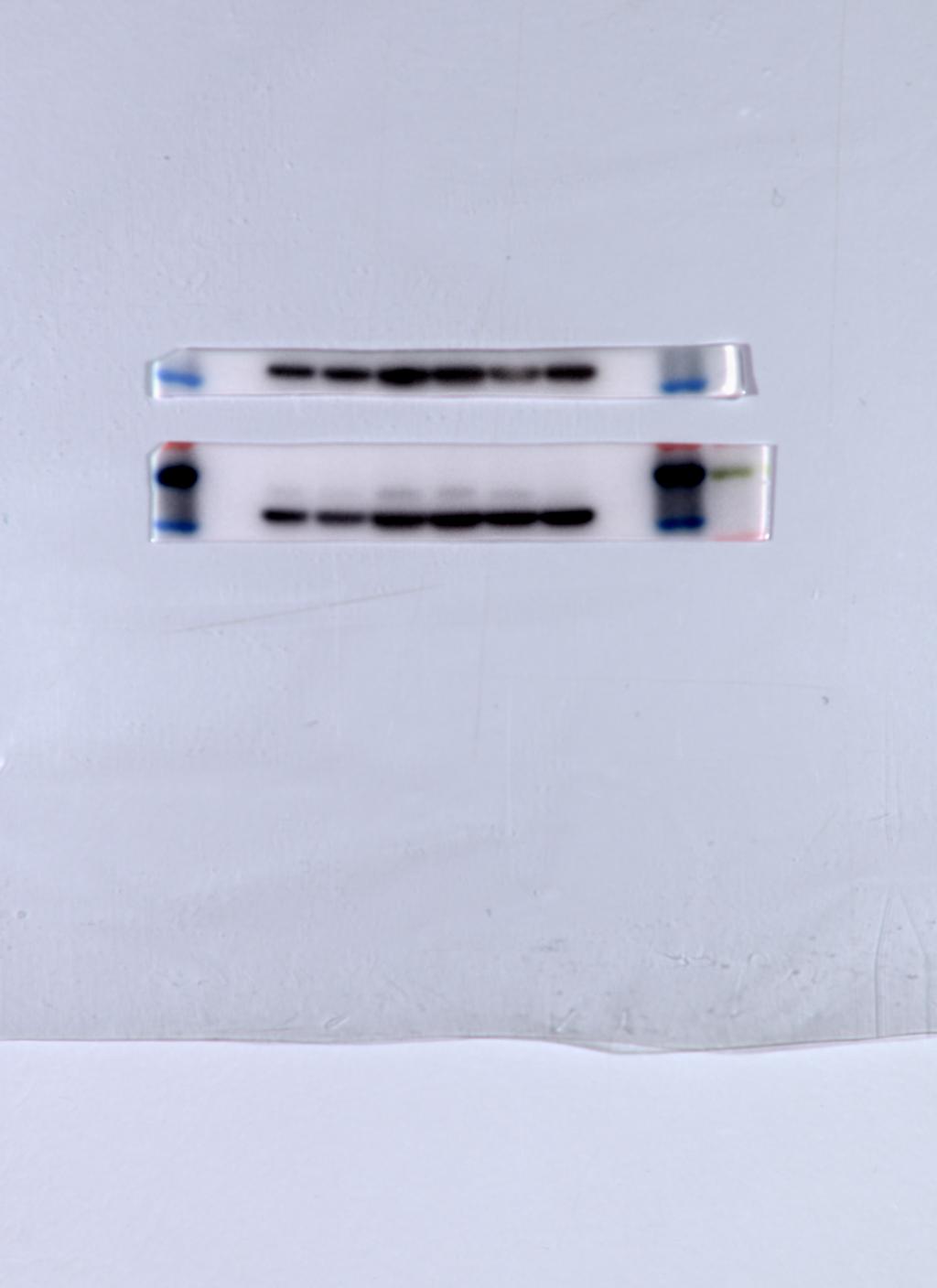

Supplement: Figure 5—figure supplement 1—source data 1. [file elife-80477-fig5-figsupp1-data1.zip › Figure 5- figure supplement 1B source data 1/1. Original files/Actin/101012 act s0.3 2019.10.12_13.12.19_Ch+Marker.jpg]

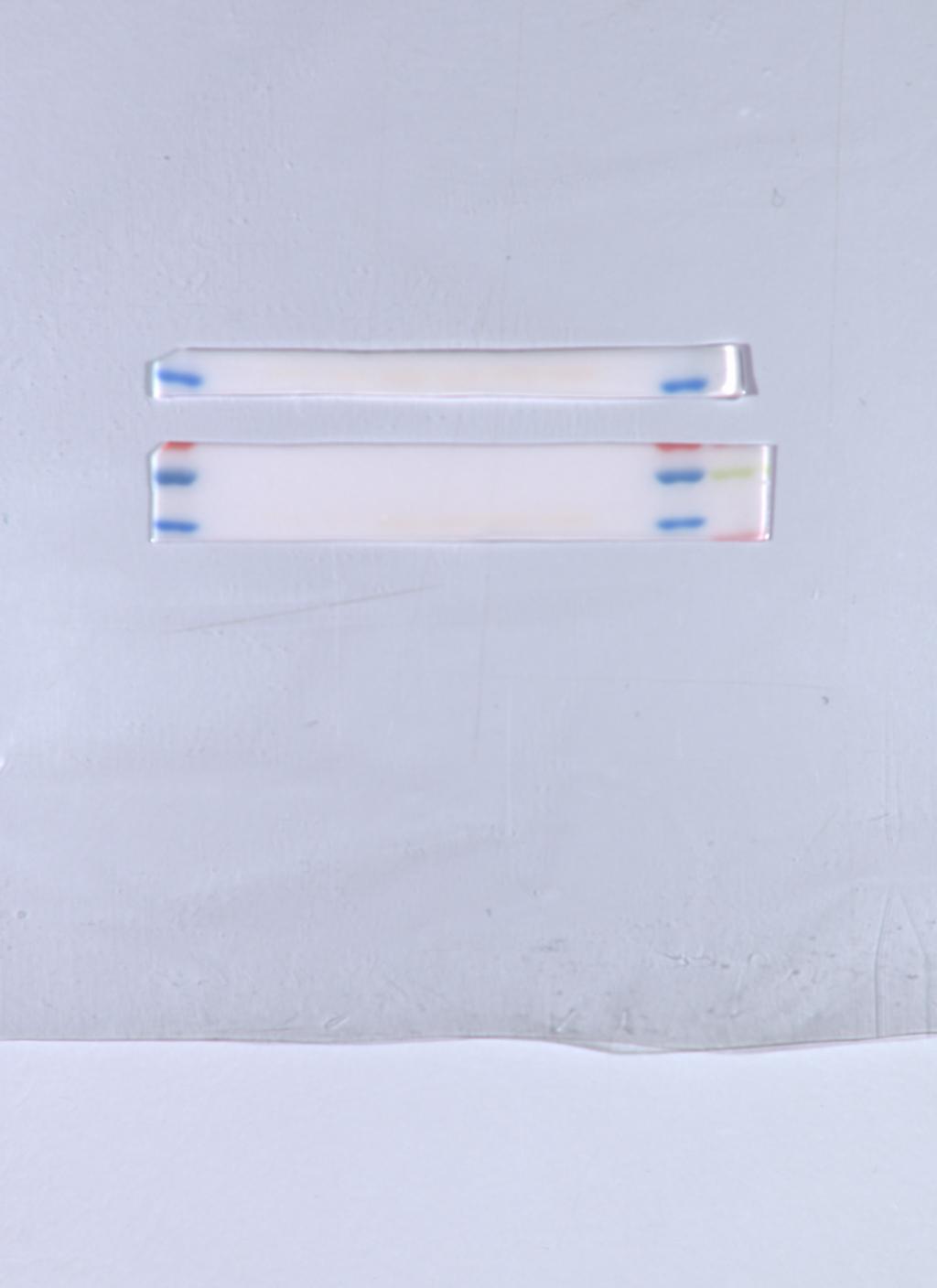

Supplement: Figure 5—figure supplement 1—source data 1. [file elife-80477-fig5-figsupp1-data1.zip › Figure 5- figure supplement 1B source data 1/1. Original files/Actin/101012 act s0.3 2019.10.12_13.12.19_Ch-Marker.jpg]

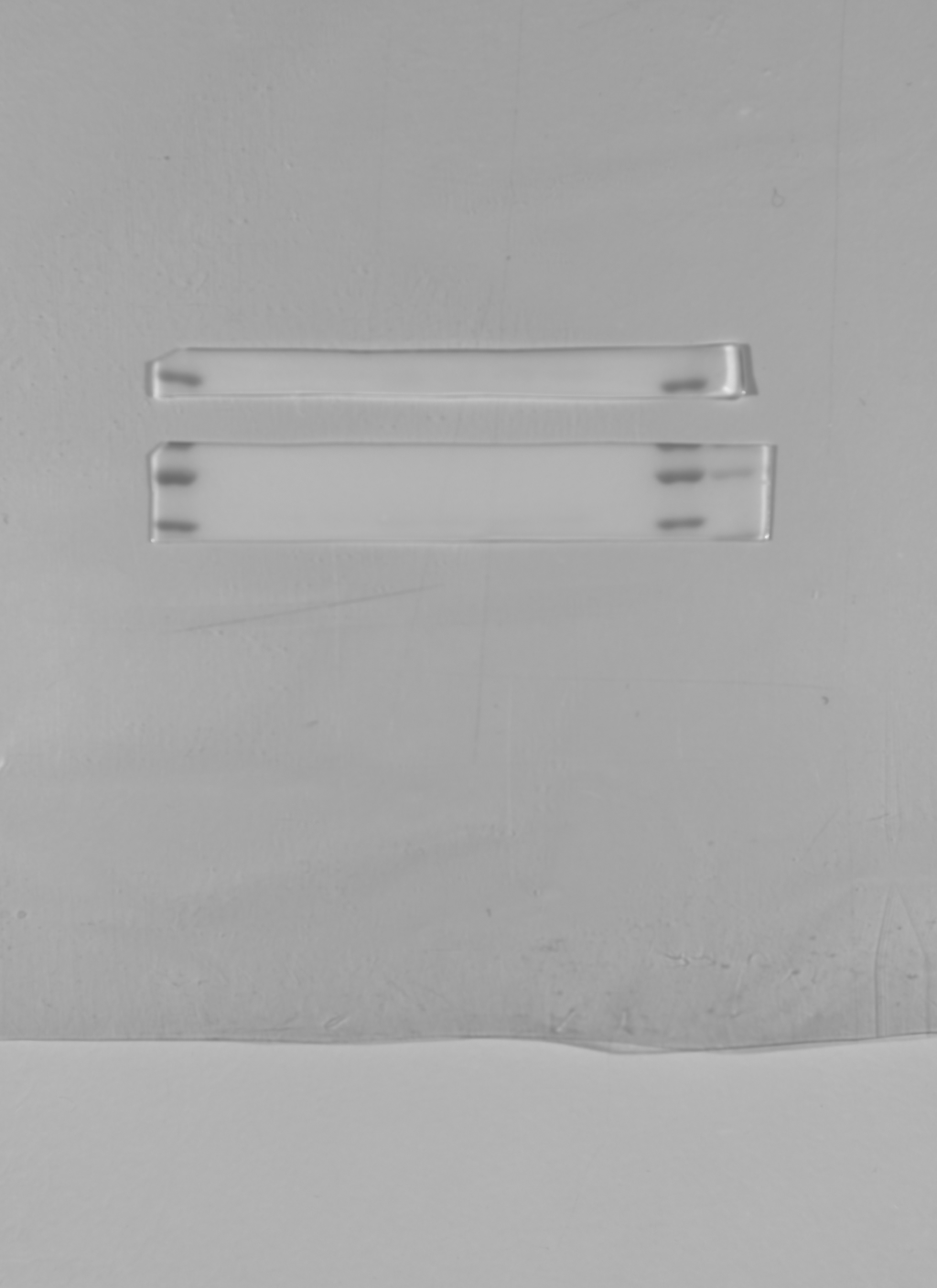

Supplement: Figure 5—figure supplement 1—source data 1. [file elife-80477-fig5-figsupp1-data1.zip › Figure 5- figure supplement 1B source data 1/1. Original files/Actin/101012 act s0.3 2019.10.12_13.12.19_Ch-Marker.tif]

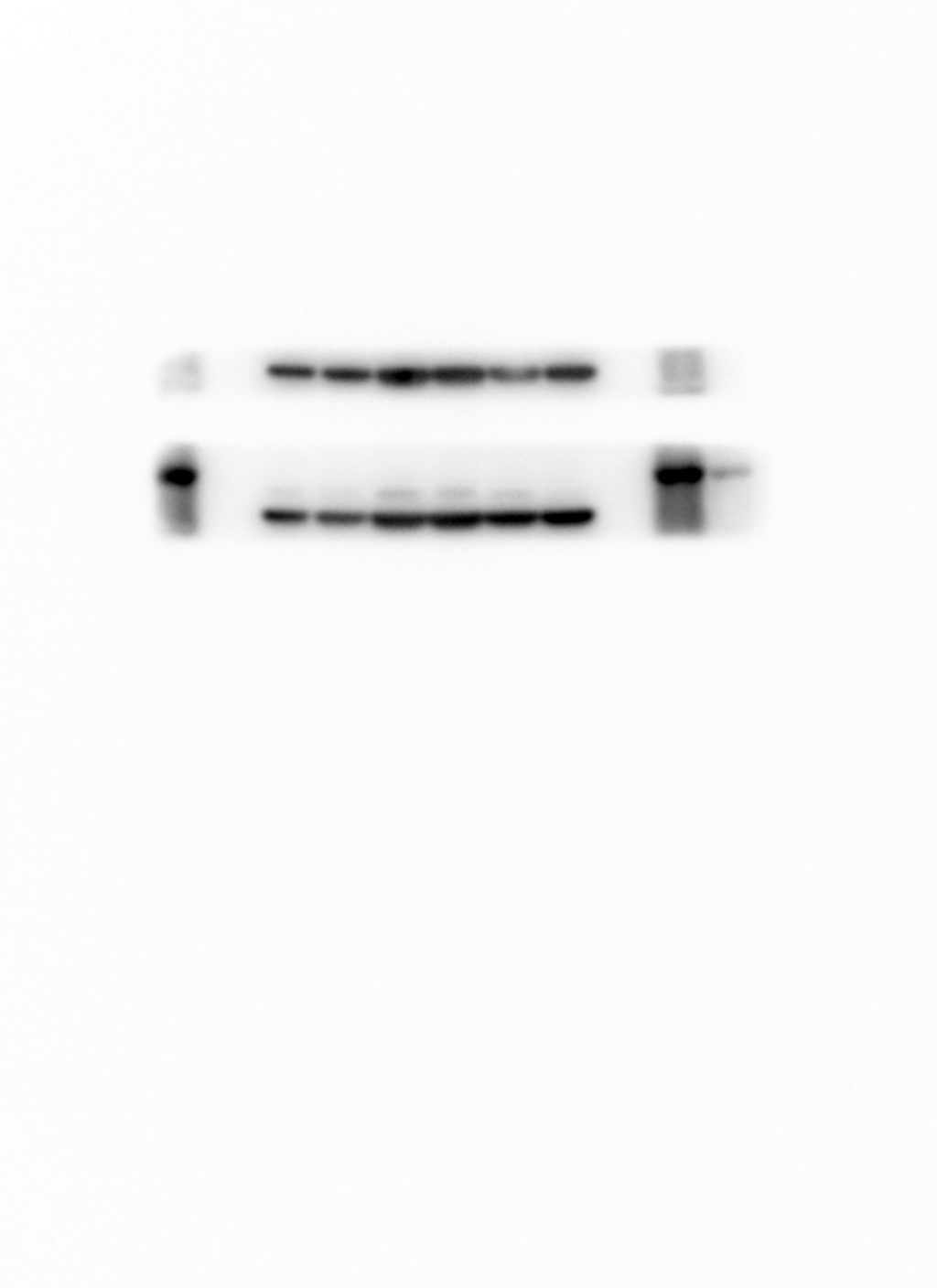

Supplement: Figure 5—figure supplement 1—source data 1. [file elife-80477-fig5-figsupp1-data1.zip › Figure 5- figure supplement 1B source data 1/1. Original files/Actin/101012 act s0.3 2019.10.12_13.12.19_Ch.tif]

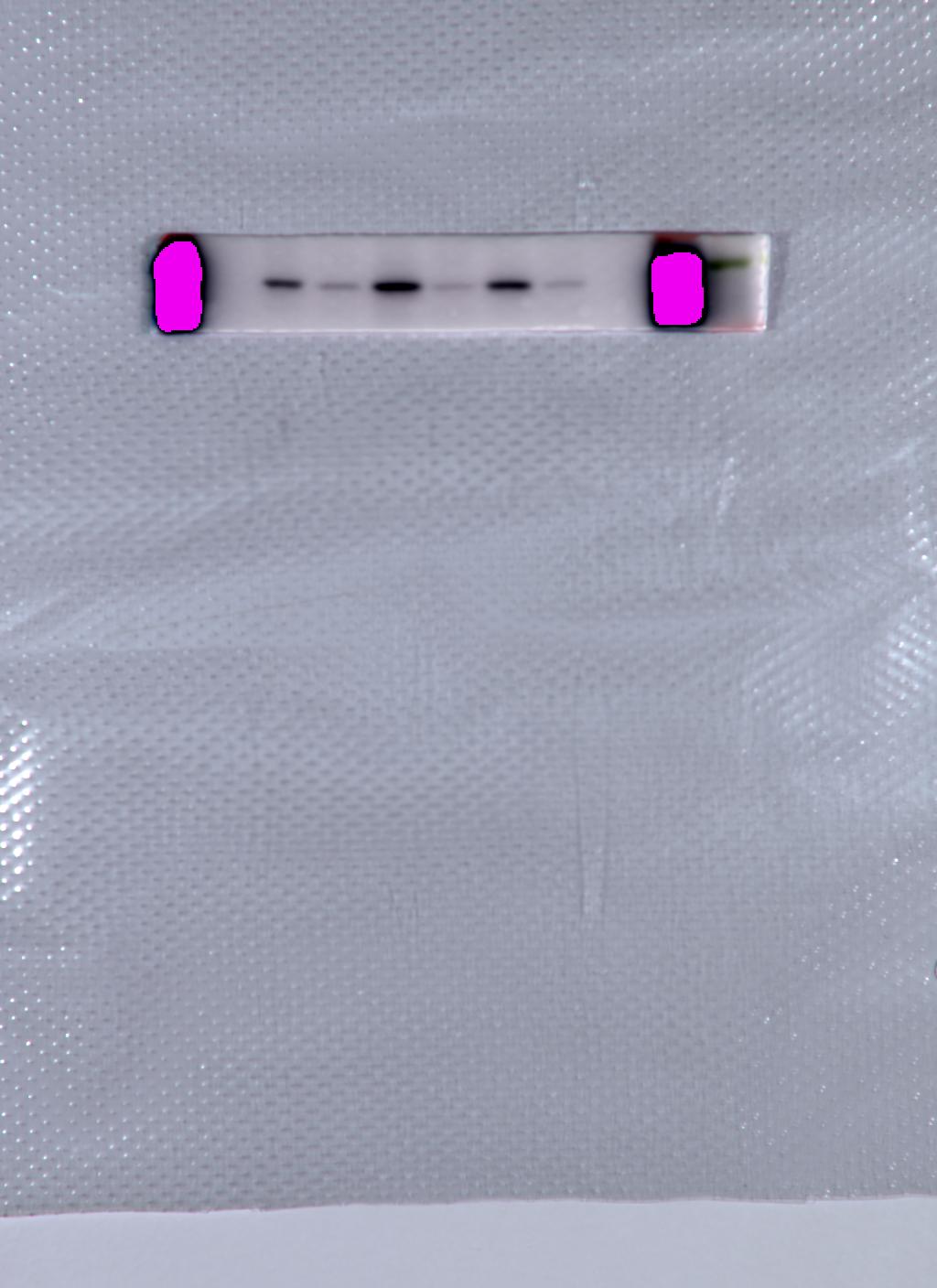

Supplement: Figure 5—figure supplement 1—source data 1. [file elife-80477-fig5-figsupp1-data1.zip › Figure 5- figure supplement 1B source data 1/1. Original files/HDAC3/191010 hdac3 s45 2019.10.10_11.38.17_Ch+Marker.jpg]

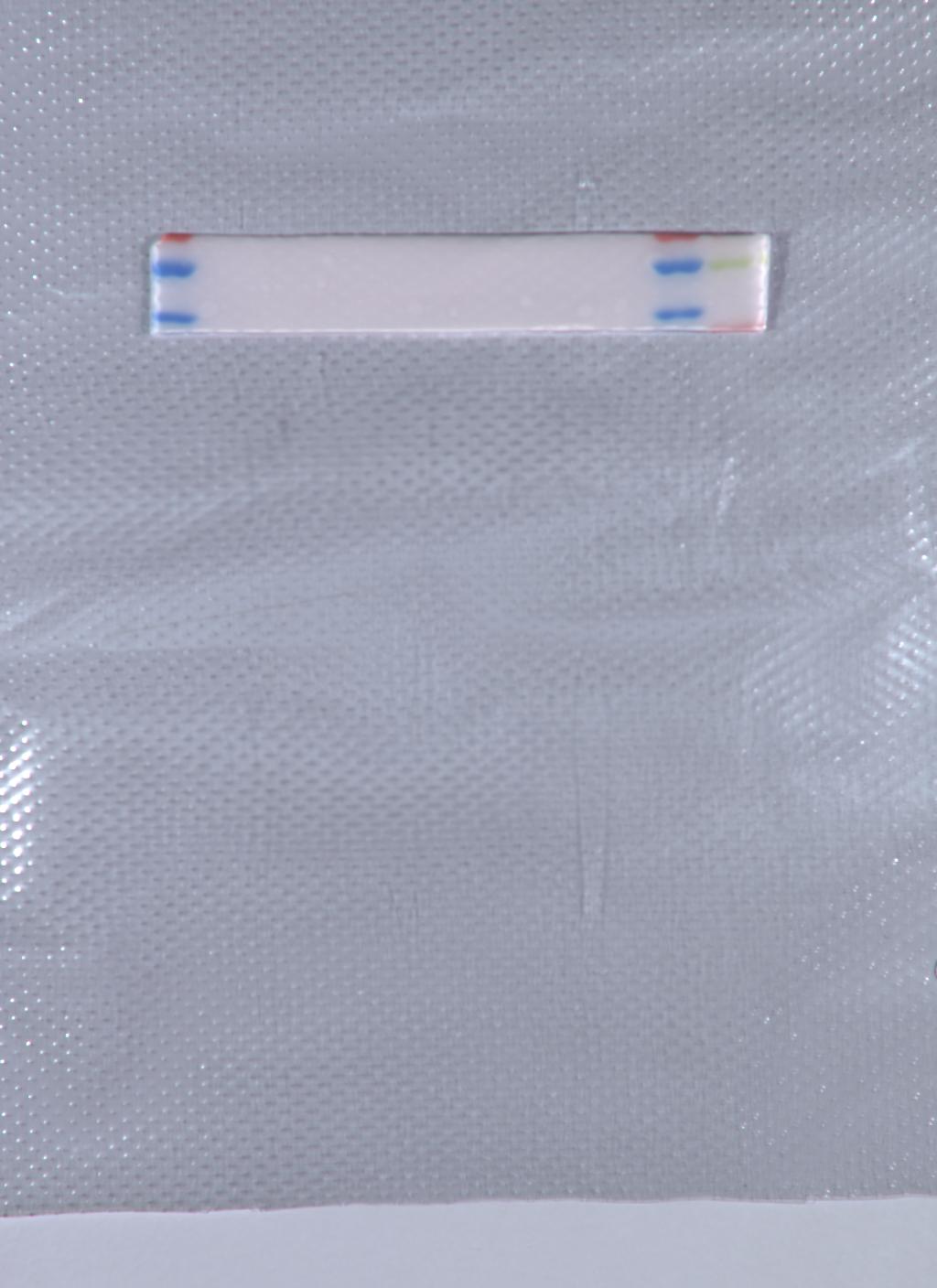

Supplement: Figure 5—figure supplement 1—source data 1. [file elife-80477-fig5-figsupp1-data1.zip › Figure 5- figure supplement 1B source data 1/1. Original files/HDAC3/191010 hdac3 s45 2019.10.10_11.38.17_Ch-Marker.jpg]

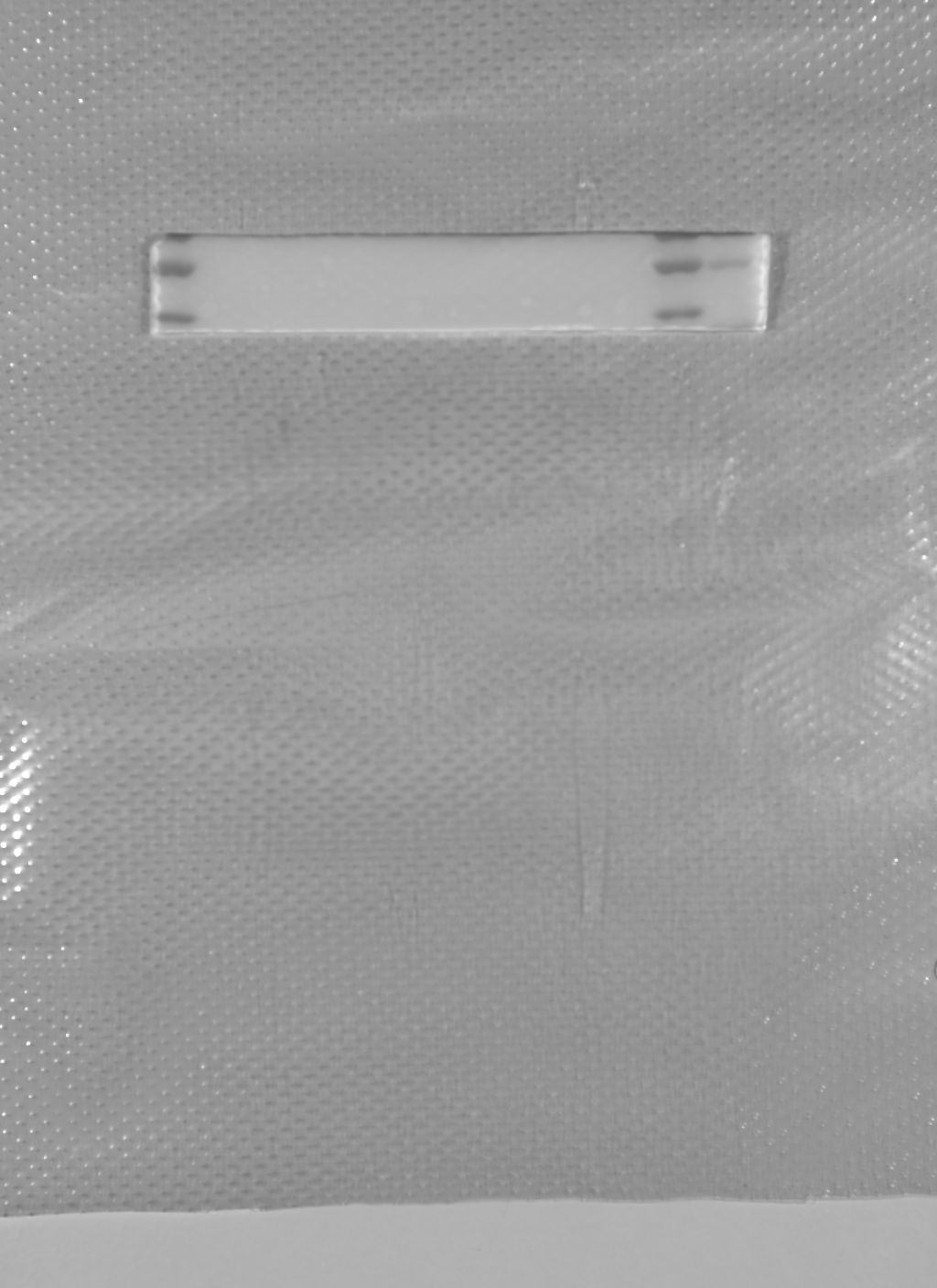

Supplement: Figure 5—figure supplement 1—source data 1. [file elife-80477-fig5-figsupp1-data1.zip › Figure 5- figure supplement 1B source data 1/1. Original files/HDAC3/191010 hdac3 s45 2019.10.10_11.38.17_Ch-Marker.tif]

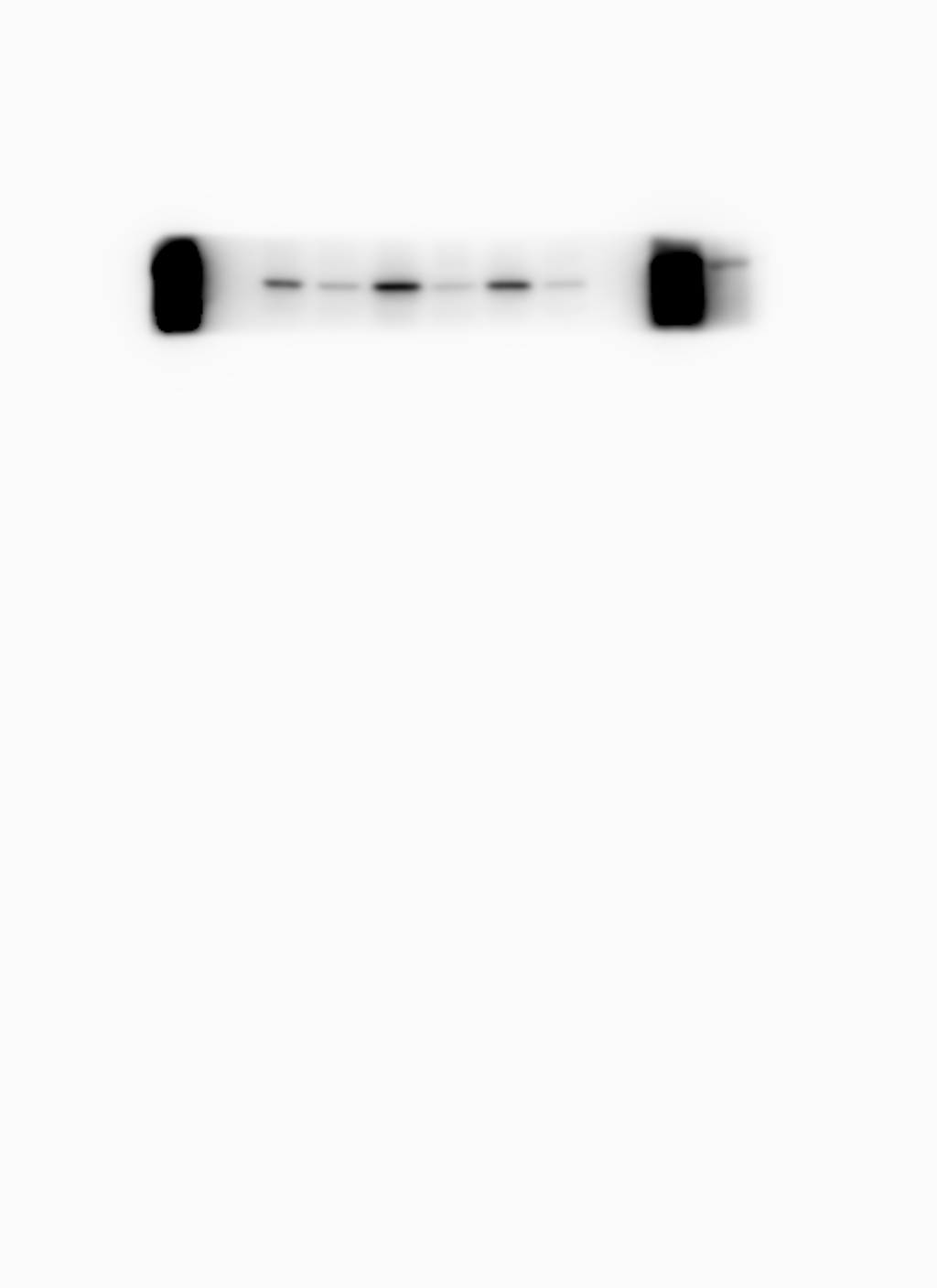

Supplement: Figure 5—figure supplement 1—source data 1. [file elife-80477-fig5-figsupp1-data1.zip › Figure 5- figure supplement 1B source data 1/1. Original files/HDAC3/191010 hdac3 s45 2019.10.10_11.38.17_Ch.tif]
