# Supplementary material for: Regulation of pDC fate determination by histone deacetylase 3
Source: eLife. 2023 Nov 27;12:e80477. doi: 10.7554/eLife.80477 (PMC10732571; doi:10.7554/eLife.80477)
Supplement: Figure 5—figure supplement 1—source data 1. [file elife-80477-fig5-figsupp1-data1.zip › Figure 5- figure supplement 1B source data 1/2. Labelled files/Figure 5- figure supplement 1B source data 1.docx]

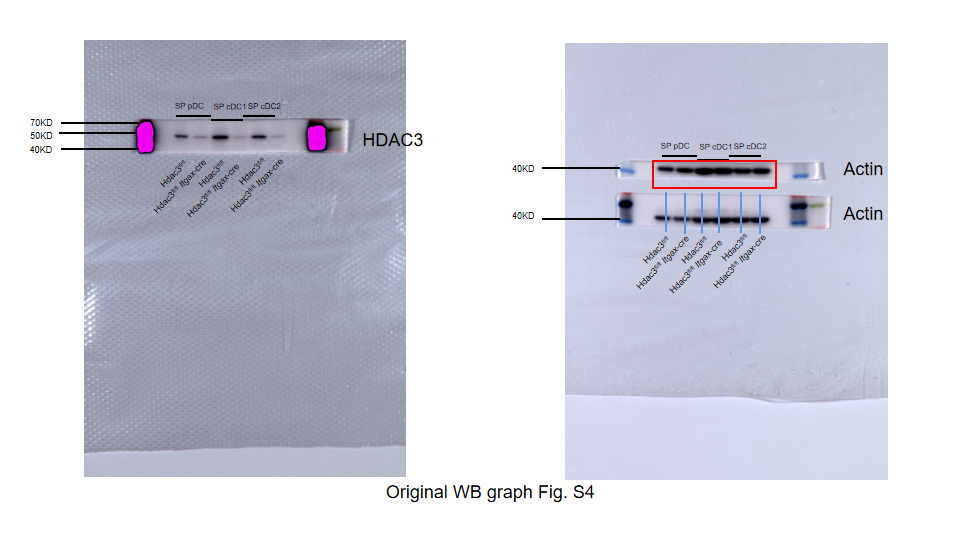


Another gel

**Figure 5 - figure supplement 1B - source data 1.** Western blot of HDAC3 knockout efficiency in *Itgax*-Cre induce HDAC3 conditional knockout splenic DC subsets.
